# Pharmacologic Ascorbate Radiosensitizes Pancreatic Cancer but Radioprotects Normal Tissue: The Role of Oxidative Stress-Induced Lipid Peroxidation

**DOI:** 10.3390/antiox13030361

**Published:** 2024-03-18

**Authors:** Gloria Y. Chen, Brianne R. O’Leary, Juan Du, Rory S. Carroll, Garett J. Steers, Garry R. Buettner, Joseph J. Cullen

**Affiliations:** 1 Departments of Surgery, Carver College of Medicine, The University of Iowa, Iowa City, IA 52242, USA; gloria-chen@uiowa.edu (G.Y.C.); brianne-oleary@uiowa.edu (B.R.O.); juan-du@uiowa.edu (J.D.); rory-carroll@uiowa.edu (R.S.C.); garett-steers@uiowa.edu (G.J.S.); 2 Free Radical and Radiation Biology Division, Department of Radiation Oncology, Carver College of Medicine, The University of Iowa, Iowa City, IA 52242, USA; garry-buettner@uiowa.edu

**Keywords:** pancreatic cancer, radioprotection, ascorbate, lipid peroxidation

## Abstract

The toxicity of ionizing radiation limits its effectiveness in the treatment of pancreatic ductal adenocarcinoma. Pharmacologic ascorbate (P-AscH^−^) has been shown to radiosensitize pancreatic cancer cells while simultaneously radioprotecting normal cells. We hypothesize that P-AscH^−^ protects the small intestine while radiosensitizing pancreatic cancer cells partially through an oxidative stress mechanism. Duodenal samples from pancreaticoduodenectomy specimens of patients who underwent radio-chemotherapy ± P-AscH^−^ and mouse tumor and jejunal samples treated with radiation ± P-AscH^−^ were evaluated. Pancreatic cancer and non-tumorigenic cells were treated with radiation ± P-AscH^−^ to assess lipid peroxidation. To determine the mechanism, pancreatic cancer cells were treated with selenomethionine or RSL3, an inhibitor of glutathione peroxidase 4 (GPx4). Radiation-induced decreases in villi length and increases in 4-HNE immunofluorescence were reversed with P-AscH^−^ in human duodenum. In vivo, radiation-induced decreases in villi length and increased collagen deposition were reversed in P-AscH^−^-treated jejunal samples. P-AscH^−^ and radiation increased BODIPY oxidation in pancreatic cancer cells but not in non-tumorigenic cells. Selenomethionine increased GPx4 protein and activity in pancreatic cancer and reversed P-AscH^−^-induced toxicity and lipid peroxidation. RSL3 treatment inhibited GPx4 activity and increased lipid peroxidation. Differences in oxidative stress may play a role in radioprotecting normal cells while radiosensitizing pancreatic cancer cells when treated with P-AscH^−^.

## 1. Introduction

Pancreatic ductal adenocarcinoma (PDAC) has persistently held a high mortality rate; with 11.1 deaths out of 100,000 cases, it is the third leading cause of cancer death in the United States. Despite advancements in research and medicine, globally, rates of survival for PDAC patients have not exceeded 10.8% [1,2]. Influenced by persistent delays in diagnosis, limited therapies and poor response to treatments, survival rates have not seen compelling improvements, but rather, the incidence and mortality are likely to increase. Radiation therapy can help to achieve local control in advance diseases through direct DNA damage as well as protein and lipid damage [3,4,5]. Most studies with radiation therapy in PDAC have not shown a significant survival benefit. However, compared to chemotherapy alone, the LAP07 trial did show improvements in local tumor progression and time to re-initiation of therapy when radiation was included as an adjuvant to chemotherapy. Radiation is also used in palliation to reduce symptoms related to progressive disease [5,6,7]. However, often, the effectiveness of radiation is limited by its toxicity to surrounding structures of the target of interest. The anatomic location of the pancreas is immediately adjacent to important organs including the small intestine and stomach that cannot be excluded from the radiation field. This leads to serious side effects such as nausea, abdominal pain, and obstruction, which result in a lower tolerance and efficacy of radiation treatment as well as a poor quality of life [8,9].

Clinical data show that when ascorbate is given orally, fasting plasma concentrations are tightly controlled at <100 µM [10,11]. In contrast, when ascorbate is administered intravenously (P-AscH^−^), plasma concentrations as high as 30 mM are safely achieved. Thus, the intravenous administration of ascorbate can yield extremely high plasma levels, while oral treatment does not. The main reason many antioxidant therapies have failed to elicit protection from free radical damage initiated by radiation is because dietary antioxidants (such as vitamin C) were used at concentrations well below pharmacological levels.

Our in vitro, in vivo [12,13,14], and human studies [11], combined with those of others [15], provide a solid foundation for using P-AscH^−^ as a radiosensitizer in PDAC therapy. P-AscH^−^ has been shown to enhance the cytotoxic effects of chemotherapies [16] and radiation in all the PDAC cell lines examined but not in non-tumorigenic pancreatic ductal epithelial cells [17,18]. In mice with established PDAC xenografts, P-AscH^−^ combined with radiation decreased tumor growth and increased survival [18]. Radiosensitization by P-AscH^−^ was associated with an increase in oxidative stress-induced DNA damage, which was reversed by catalase [18]. Also, P-AscH^−^ reversed radiation-induced damage to the jejunum and did not increase systemic changes in parameters indicative of oxidative stress [17]. These very encouraging studies led us to perform the first-in-human phase I trial with gemcitabine, ascorbate, and radiation therapy for PDAC (NCT01852890). This trial demonstrated that P-AscH^−^ in combination with gemcitabine and radiation for locally advanced PDAC was safe, well tolerated, and showed suggestions of efficacy [17]. The median overall survival of patients treated with P-AscH^−^, gemcitabine, and radiation increased compared to the comparator arm of the study (*p* = 0.05) as well as the historical median overall survival [19].

These results clearly demonstrate the potential clinical utility of P-AscH^−^ as a radiosensitizer in the treatment of PDAC. While previous research has focused on whether P-AscH^−^ enhances the effectiveness of radiation, there is additional evidence to suggest that it may also play a role in protecting normal cells from radiotherapy [17]. The aims of our study were to determine if P-AscH^−^ demonstrated radioprotectant qualities in normal human and animal tissues. We also wanted to determine potential mechanisms of radiosensitization in PDAC cells. Our novel study indeed demonstrates radioprotection in an animal model; however, most exciting is that P-AscH^−^ also demonstrates the protection of the small intestine in humans. We also determined that these differences may be due to increased oxidative stress in tumor cells, whereas in normal cells, the actions of glutathione peroxidase 4 lead to decreased lipid peroxidation, i.e., less oxidative stress.

## 2. Materials and Methods

### 2.1. Patient Sample and Staining

This clinical trial was conducted under an established investigator-sponsored IND. Compliant with ICMJE policy, the trial was listed on clinicaltrials.gov prior to enrollment (NCT01852890). Approval was obtained from the University of Iowa IRB. The trial was conducted under Good Clinical Practice consistent with the International Council on Harmonization guidance document (ICH E6(R2)). Subjects who subsequently underwent pancreaticoduodenectomy received P-AscH^−^, 75 g in 1000 mL IV. P-AscH^−^ was infused daily. Gemcitabine was administered according to ECOG-E4201 with an intravenous infusion at a dose of 600 mg/m^2^ over 30 min, once weekly for six weeks [19]. Intensity-modulated radiation therapy (IMRT) was delivered with either Siemans Oncor (Malvern, PA, USA) or Elekta Versa HD (Stockholm, Sweden) treatment machines as either 50.4 Gy in 28 fractions or 50 Gy in 25 fractions as determined most appropriate by the treating radiation oncologist. Patient duodenal and tumor samples were provided by the Biospecimen Procurement and Molecular Epidemiology Resources (BioMER) at the University of Iowa Holden Comprehensive Cancer Center (Iowa City, IA, USA). Patients who underwent the same gemcitabine and radiation combination and then underwent a pancreaticoduodenectomy were used as controls. Slides were prepared by the University of Iowa Department of Pathology and stained with hematoxylin and eosin as well as 4-hydroxynonenal (4-HNE) antibodies. Villi length was then measured similarly as described below. Levels of 4-HNE immunofluorescence staining was measured by calculating the ratio of the percent area of 4-HNE to DAPI using ImageJ 1.52d (National Institutes of Health; Bethesda, MD, USA).

### 2.2. In Vivo Orthotopic Mouse Experiment

Animal protocols were reviewed and approved by the Animal Care and Use Committee of the University of Iowa. Female C57BL/6J mice were purchased from Envigo. The mice received pancreatic injections of 1 × 10^6^ PANC-02luc cells in Corning Matrigel^®^ (Burlington, MA, USA), luciferase-expressing mouse PDAC cell lines. After tumor formation (10 d after injection), the mice were divided into four treatment groups: saline alone, saline and radiation, P-AscH^−^ alone, and P-AscH^−^ and radiation. The mice were treated with twice-daily injections of intra-peritoneal (I.P.) saline (1 M) or P-AscH^−^ (4 g/kg). A radiation dose of 8 Gy was given on day 4 and 10 of treatment. Tumor growth was monitored periodically over the course of the treatment using bioluminescent imaging to determine the tumor burden. Tumors and jejunal tissue were harvested 25 days after tumor cell injection and processed for analyses. Mouse jejunal and tumor sample slides were prepared by the University of Iowa Department of Pathology and stained with hematoxylin and eosin in addition to trichrome staining. Slides were converted into electronic images. Using ImageJ, the villi length was measured as the distance from the tip of the villi to the submucosa in micrometers. The collagen volume fraction was determined by the ratio of blue-stained area to entire tissue area.

### 2.3. Cell Culture

MIA PaCa-2 and PANC-1 human PDAC cell lines were obtained from ATCC (Manassas, VA, USA) and cultured in Dulbecco’s Modified Eagle’s Media (DMEM) supplemented with 10% fetal bovine serum (FBS) and 1% penicillin–streptomycin antibiotic. PANC-02luc mouse cells were a kind gift from Dr. Edward Filardo and cultured in DMEM supplemented with 10% FBS. The patient-derived PDAC cell line PDX-339 was obtained from the Medical College of Wisconsin surgical oncology tissue bank [20,21] and cultured in DMEM/F-12 media supplemented with 10% FBS, insulin, EG, hydrocortisone, bovine pituitary extract, and 1% penicillin–streptomycin antibiotic. The non-tumorigenic pancreatic ductal epithelial cell line H6c7 was purchased from Kerafast^®^, Inc. (Boston, MA, USA) and cultured in keratinocyte serum-free media supplemented with epidermal growth factor (5 ng/mL) and bovine pituitary extract (50 µg/mL). Three-day normal human fibroblasts were obtained from the Coriell Institute for Medical Research (Camden, NJ, USA), and the immortal human umbilical vein endothelial cell line EA.hy926 was obtained from ATCC. Both cell lines were cultured in DMEM with 10% FBS and 1% penicillin–streptomycin antibiotic. HPSCs, an immortalized tumor-associated pancreatic stellate cell line, were obtained from Hwang et al. and maintained in DMEM with 10% FBS and 1% penicillin–streptomycin [22,23].

### 2.4. Reagent/Drug and Ionizing Radiation Treatment

A stock solution of 1.00 mol/L, pH 7 L-ascorbic acid was used for ascorbate treatments. This solution was prepared under argon and subsequently stored at 4 °C in glass vials with a tight-fitting stopper. The concentration of the ascorbate was verified prior to use at 265 nm, ε_265_ = 14,500 M^−1^ cm^−1^ [24]. Ascorbate treatments for all cell types were carried out in fresh 10% DMEM media for 1 h. Doses of ascorbate were calculated as moles per cell [25]. Cells were treated with ascorbate 1 h prior to radiation.

Cells were treated with catalase at 100 µg/mL immediately prior to treatment with ascorbate. Selenomethionine (200 nM) treatment was given for 48 h and removed prior to the addition of other treatments. RAS-selective lethal 3 (RSL3) (Cayman; Ann Harbor, MI, USA, #19288), an inhibitor of glutathione peroxidase 4 (GPx4), was given 3 h prior to isolation or fluorescent staining at a concentration of 50–200 nM. Ascorbate was then added directly to the media after 1 h of RSL3 incubation. Cells were radiated in the Iowa Radiation and Free Radical Research Core facility using a cesium-137 gamma radiation source for a total of 2–6 Gy.

### 2.5. Western Blotting

Cells were isolated and protein samples prepared with phosphosafe buffer or RIPA lysis buffer. Protein concentrations were determined utilizing the Bradford protein assay. A total of 20–40 µg of protein were electrophoresed with a 4–20% SDS-PAGE gradient gel. Proteins were electro-transferred onto nitrocellulose membrane and blocked with 5% nonfat milk in 0.1% Tween-PBS (TPBS). Primary antibodies catalase (Cell Signaling; Danvers, MA, USA, #14097S, 1:4000) and GPx4 (Abcam; Waltham, MA, USA, #ab125066, 1:6000) were used to incubate membranes at 4 °C overnight. The membranes were then incubated in the appropriate horseradish peroxidase-conjugated secondary antibodies at 1:10,000 to 1:20,000 concentrations. GAPDH (Cell Signaling; #D16H11) with a primary concentration of 1:5000 and secondary concentration of 1:50,000 or Actin (EMD Millipore; Burlington, MA, USA, #MABT825) at a primary concentration of 1:4000 and a secondary concentration of 1:20,000 were used as loading controls. After being washed with TPBS, the membranes were visualized by staining with SuperSignal West Pico (PLUS) substrate and exposing with classic blue autoradiography film. Mouse tumors and jejunal tissue were processed to determine 4-hydroxy-2-nonenal (4-HNE, Invitrogen; Waltham, MA, USA, #: MA5-27570)-modified proteins as previously described [18,20,26,27]. After the normalization of the 4-HNE immunoreactive protein-to-protein loading, an analysis was performed with average densities determined with ImageJ.

### 2.6. Clonogenic Survival Assay

Cell culture treatments were performed by adding P-AscH^−^ doses with and without selenomethionine in various combinations as described above. After 1 h of treatment with P-AscH^−^, cells were radiated at 4 Gy. Immediately after treatment, the cells were trypsinized and counted using a Countess II automated cell counter. The cells were then plated into each well in triplicate with a specific number of cells depending on treatment type in their respective medium. After 10–14 days, the cells were fixed with ethanol and stained with Coomassie blue. Colonies greater than 50 cells were counted and the surviving fraction calculated by the number of colonies/number of cells seeded and normalized to a control for each condition. Each experiment was performed in no less than *n* = 3 biological replicates.

### 2.7. Measurement of Lipid Peroxidation

Lipid peroxidation was measured via confocal microscopy using fluorescent marker BODIPY C-11^581/591^ (ThermoFisher Scientific; Rockford, IL, USA, #D3861). Media were removed from live cell samples 1 h after radiation treatment and washed twice with phosphate-buffered saline (PBS). BODIPY C-11 was diluted to 1 µM solution with phenol-free media with 10% FBS. Dishes were stained with the BODIPY solution and incubated for 30 min. Plates were washed again prior to adding 4 mL PBS. The submersible objective lens of the confocal microscope was used to the measure emission of the reduced and oxidized forms of the BODIPY probe. *Tert*-butyl hydroperoxide was used as a positive control at 500 µM. This was carried out in triplicate for each plate. ImageJ was used to quantify the ratio of the oxidized to reduced area and each normalized to the control sample.

### 2.8. Activity Assays, Catalase and GPx4

Catalase activity was measured via a UV-Vis spectrophotometer determining the rate of removal of hydrogen peroxide after the addition of catalase as described previously [28,29]. The activity of catalase in cells was determined as m*k*Units per million cells.

GPx4 activity was measured via a spectroscopic kinetic assay developed by and previously described by Stolwijk et al. [30]. Cells (3–6 × 10^6^) were collected via trypsinization and washed with PBS; cell pellets were stored at −80 °C. Cell pellets were resuspended in 200 µL GPx assay buffer (100 mM Tris base, pH 8.0, 1.5 mM NaN_3_, 2.0 mM EDTA and 0.1% Triton X-100). After centrifugation at 10,000× *g* for 10 min, 50 µL supernatant were added to 1 mL (1 cm pathlength) quartz cuvette, followed by 50 µL each of 4 mM NADPH, 30 U mL^−1^ glutathione disulfide reductase and 60 mM glutathione, and 785 µL assay buffer. The assay mixture was incubated at 37 °C for 5 min. The loss of NADPH absorbance at 340 nm was then followed for about 300 s after the addition of H_2_O_2_. The rate of NADPH oxidation (slope) for the background and with H_2_O_2_ were determined. The extinction coefficient ε_340_ = 6270 M^−1^ cm^−1^ at 37 °C and pH 8 was used to calculate the activity of GPx4. GPx4 activity was expressed as mU mg^−1^ protein.

### 2.9. Glutathione Levels

Total glutathione was measured by a plate reader assay [31]. Cells (1–2 × 10^6^) were harvested via centrifugation. Cell pellets were resuspended in 200 µL 0.1 M sodium phosphate buffer (pH 7.5) containing 0.1% Triton x-100 and 0.6% 5-sulfosulicylic acid (SSA) and stored at −80 °C. After centrifugation at 16,000× *g* in a microcentrifuge, 20 µL cell lysate was added to the wells of a 96-well plate, followed by 120 µL of glutathione reductase (GR) and 5,5′-dithio-bi-(2-nitrobenzoic acid) (DTNB) mixture, and then 60 µL of NADPH was added to the reaction mixture. The final concentrations of reagents were 0.25 U mL^−1^ GR, 0.5 µM DTNB, and 0.2 mM NADPH. The reaction rate of DTNB reduction was monitored at 412 nm for 2 min with 20 s interval. Total glutathione concentration expressed as mM were calculated from a glutathione standard curve (ranging from 0.002 to 0.52 nmoL) and the cell volume measured with a Moxi Zautomated cell counter (Oroflo, Ketchum, ID, USA).

### 2.10. Statistical Methods

Statistical analysis of two groups was carried out with unpaired 2-tailed Student’s *t*-tests. One-way ANOVA analysis was utilized for multiple comparisons using the Tukey’s multiple-comparisons test. The data are presented as mean ± SEM. All experiments were completed at least in triplicates. GraphPad Prism^®^ (version 10, La Jolla, CA, USA) was used to perform all analyses.

## 3. Results

### 3.1. P-AscH^−^ Ameliorates Radiation-Induced Intestinal Damage

**Figure 1:**
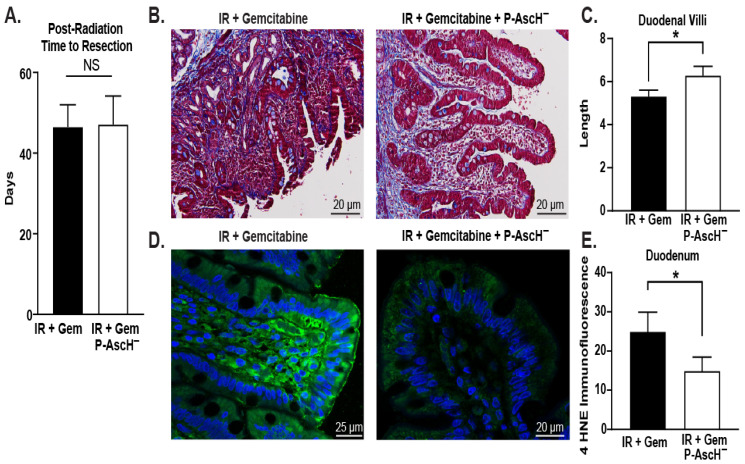
Duodenal samples were obtained from pancreaticoduodenectomy specimens of patients who received radiation and gemcitabine with or without P-AscH^−^. (**A**) There were no differences in days to resection after treatment (*n* = 3 per group, means ± SEM, unpaired *t*-test, *p* = 0.94). (**B**) Representative slides (20 µm) of duodenal samples stained with trichrome showing the protection afforded by P-AscH^−^. (**C**) Average villi length was increased in duodenal samples from patients treated with P-AscH^−^ (IR + Gem *n* = 22, IR + Gem + P-AscH^−^ *n* = 19, means ± SEM, unpaired *t*-test, * *p* ≤ 0.05). (**D**) Duodenal samples stained with 4-HNE immunofluorescence antibody indicating less lipid peroxidation with P-AscH^−^ (20–25 µm). (**E**) Quantification of 4-HNE immunofluorescence (**D**) as percent area of image was decreased in duodenal samples from patients treated with P-AscH^−^ (means ± SEM, IR + Gem *n* = 10, IR + Gem + P-AscH^−^ *n* = 15, Mann–Whitney test, * *p* ≤ 0.04).

It has been demonstrated that PDAC patients treated with radiation and gemcitabine with P-AscH^−^ vs. radiation and gemcitabine alone demonstrated increases in the overall and progression-free survival [17]. Duodenal samples were obtained from pancreaticoduodenectomy specimens in patients who underwent radiation or gemcitabine with P-AscH^−^ and compared to patients who had the same operation after treatment with radiation or gemcitabine alone (controls). The time to resection following the completion of preoperative chemo-radiation were similar in both groups (Figure 1A). Slides of duodenal samples were stained with trichome and evaluated for villi length demonstrating that treatment with P-AscH^−^ increased villi length compared to the controls (Figure 1B and quantified in Figure 1C). A 4-hydroxynonenal (4-HNE) immunofluorescence antibody was used to stain the samples to evaluate protein oxidation, a marker of oxidative stress. Duodenal samples from patients treated with radiation, gemcitabine, and P-AscH^−^ had decreased the staining of 4-HNE compared to the controls (Figure 1D,E). The preservation of villi length and decreased oxidation show protective effects of P-AscH^−^ on the small bowel adjacent to the pancreas undergoing radiation.

**Figure 2:**
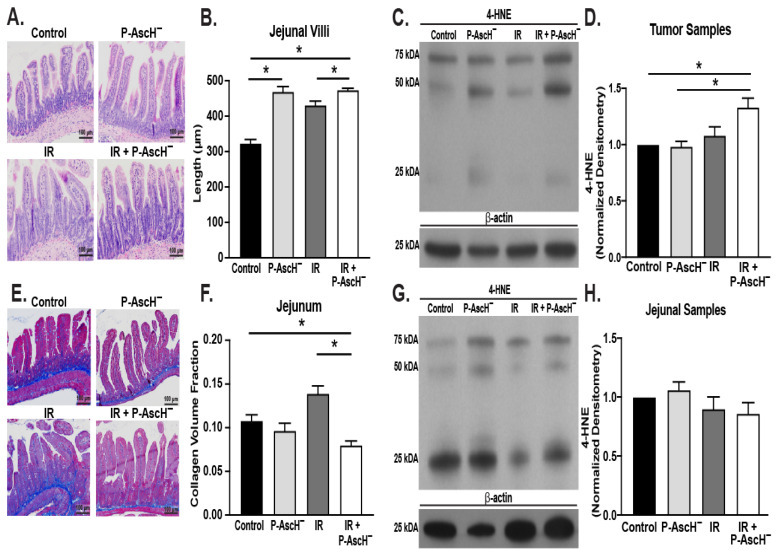
Orthotopic PDAC tumors models in C57BL/6J black mice injected with PANC02Luc cells. Intraperitoneal P-AscH^−^ or saline treatments were started after tumor formation was confirmed by bioluminescent imaging. Radiation (8 Gy) was given on days 4 and 10. Tumors and jejunal tissue were removed 26 days post tumor injection. (**A**) Representative jejunal tissue slides stained with H&E (100 µm). (**B**) P-AscH^−^ and radiation increased average jejunal villi length compared to radiation alone (means ± SEM, *n* = five–eight mice per group with two–four slides per mouse, unpaired *t*-test, * *p* ≤ 0.05). (**C**) Representative Western blot image of 4-HNE in protein samples isolated from tumor tissue. Β-actin was used a loading control. (**D**) Radiation-induced increases in collagen volume fraction was reversed with the addition of P-AscH^−^ (means ± SEM, *n* = five mice per group, unpaired *t*-test, * *p* ≤ 0.05). Quantification of 4-HNE protein expression in tumor samples demonstrated increases in 4-HNE with the combination of radiation and P-AscH^−^ compared to control. (**E**) Representative jejunal samples stained with trichrome (100 µm). (**F**) Radiation-induced increases in collagen volume fraction was reversed with the addition of P-AscH^−^ (means ± SEM, *n* = five–eight mice per group with two–four slides per mouse, unpaired *t*-test, * *p* ≤ 0.05). (**G**) Representative Western blot image of 4-HNE in protein samples isolated from jejunal tissue. Β-actin was used a loading control. (**H**) Quantification of protein concentration in jejunal samples demonstrating no significant changes in 4-HNE protein expression among the various treatment groups (*n* = five mice per group).

These results were then further expanded using an orthotopic mouse model where immunocompetent mice underwent intrapancreatic injections of PANC02Luc cells (catalase activity: 4.7 m*k*U mg^−1^). The mice were radiated with ± P-AscH^−^, and both tumor tissue and jejunal tissue were removed. Similar to patient results, P-AscH^−^ induced radioprotection as seen by the increased average villi length compared to radiation alone (Figure 2A and quantified in Figure 2B). Additionally, the average collagen volume fraction was decreased with the addition of P-AscH^−^, consistent with decreased fibrosis (Figure 2E). As seen in Figure 2F, the radiation-induced increase in the collagen volume fraction was reversed with the addition of P-AscH^−^.

Previous studies from our group have demonstrated that P-AscH^−^ radiosensitizes tumor tissue and may radioprotect normal tissues [17]. The combination of P-AscH^−^ and radiation in tumor tissue (Figure 2C) increased 4-HNE staining compared to no treatment and treatments with P-AscH^−^ alone which is quantified in Figure 2D. 4-HNE was then evaluated in jejunal samples (Figure 2G) in the radiation field, demonstrating no significant changes in any of the treatment groups, as seen in Figure 2H.

### 3.2. Lipid Peroxidation Contributes to P-AscH^−^-Induced Radiosensitivity

**Figure 3:**
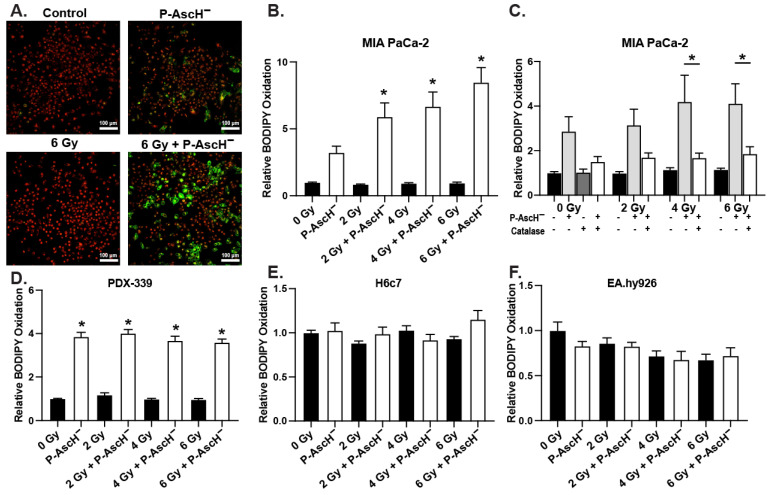
PDAC and normal cells were radiated with 2–6 Gy and treated with or without 1 mM (10–20 picomoles cell^−1^) P-AscH^−^. Cells were then incubated with BODIPY C-11 for 30 min and evaluated under confocal microscopy. (**A**) Representative confocal images (100 µm) of MIA PaCa-2 cells where red = reduced, green = oxidized. (**B**) Quantified relative BODIPY oxidation of MIA PaCa-2 cells shows a dose-dependent increase in lipid peroxidation following treatment with P-AscH^−^ (means ± SEM, * *p* ≤ 0.05 vs. radiation alone, *n* = 3). (**C**) BODIPY oxidation of MIA PaCa-2 cells is decreased upon treatment with catalase in the medium (100 µg, means ± SEM, * *p* ≤ 0.05 vs. catalase + P-AscH^−^, *n* = 3). (**D**) BODIPY oxidation increases in PDX-339 PDAC cells following P-AscH^−^ treatment (means ± SEM, * *p* ≤ 0.05 vs. radiation alone, *n* = 3). (**E**) No changes in BODIPY oxidation are seen in non-tumorigenic H6c7 cells treated with radiation and P-AscH^−^ (means ± SEM, *n* = 3). (**F**) No changes in BODIPY oxidation are seen in the human umbilical vein cells EA.hy926 treated with radiation and P-AscH^−^ (means ± SEM, *n* = 3).

Both preclinical in vivo studies and patient-derived samples indicate that P-AscH^−^ radiosensitizes tumor tissue but may radioprotect normal tissue. We hypothesized that lipid peroxidation may contribute to these differential effects. MIA PaCa-2 cells were exposed to 2–6 Gy of IR and treated with or without P-AscH^−^. In MIA PaCa-2 cells, BODIPY C-11 fluorescence, an indicator of lipid peroxidation, was increased in cells treated with P-AscH^−^ compared to radiation alone (Figure 3A). When BODIPY fluorescence was quantified, there was a radiation-induced dose-dependent increase in lipid peroxidation with radiation alone, as seen in Figure 3B. With the addition of catalase, there was a partial reversal of the oxidation seen with BODIPY staining (Figure 3C), suggesting a P-AscH^−^ increased lipid peroxidation in these cancer cells.

Radiation with P-AscH^−^ also increased BODIPY fluorescence in the patient-derived PDAC cell line PDX-339. However, this did not to appear to be dose-dependent on P-AscH^−^ (Figure 3D). The increases in BODIPY fluorescence seen in the tumor cells treated with radiation and P-AscH^−^ were not seen in the non-tumorigenic pancreatic ductal epithelial cell line H6c7 (Figure 3E) and the non-tumorigenic endothelial cell line EA.hy926 (Figure 3F). While there is a component of a hydrogen peroxide-associated mechanism in the radiosensitivity of P-AscH^−^, this had not manifested in the oxidation of BODIPY in the non-tumorigenic cell lines. Cancer cells often have lower levels of catalase compared to normal cells [32]. However, the protein levels (Supplemental Figure S1A) and activity levels (Supplemental Figure S1B) of catalase in this set of cancer cells were not significantly different from the non-tumorigenic cells studied, PANC-1 cells being an exception, which have higher levels of catalase activity than non-tumorigenic cells in this study. Thus, differences in sensitivity appear not to be due to different levels of catalase.

### 3.3. Selenomethionine Reverses the Effects of P-AscH^−^ on PDAC Cells

**Figure 4:**
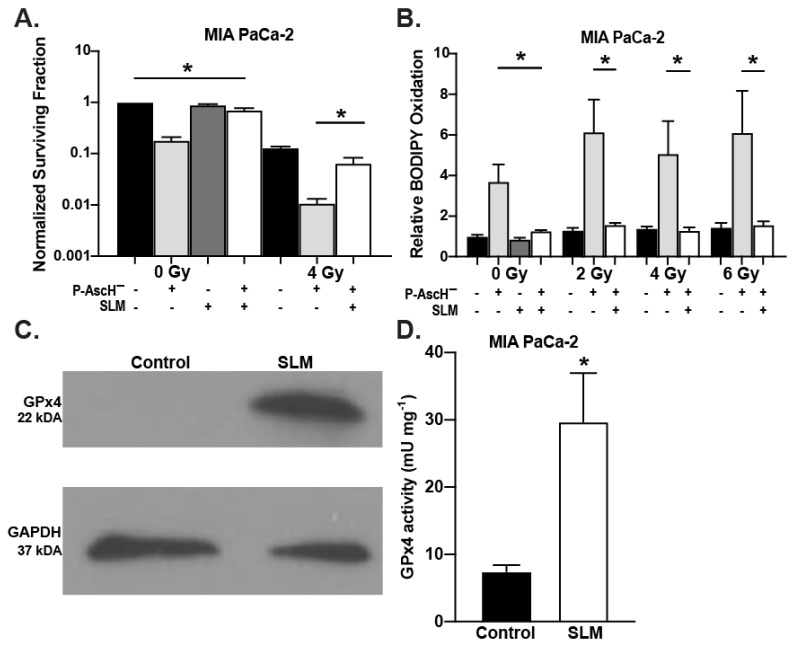
MIA PaCa-2 cells were radiated (2–6 Gy) and treated with 1 mM P-AscH^−^ (1 h, 10 picomoles cell^−1^), selenomethionine (SLM) (8 h, 200 nM), or in combination. (**A**) Clonogenic survival was decreased following radiation, P-AscH^−^, or radiation plus P-AscH^−^ treatment. SLM treatment reversed the effects of P-AscH^−^. (means ± SEM, *n* = 3, * *p* ≤ 0.05 vs. P-AscH^−^). (**B**) Cells were then incubated with BODIPY C-11 for 30 min after treatment and evaluated under confocal microscopy. SLM reverses effect of P-AscH^−^ on BODIPY oxidation. Data represent relative BODIPY oxidation (means ± SEM, * *p* ≤ 0.05, two-way ANOVA, *n* = 3). (**C**) Representative Western blot image of GPx4 following treatment with SLM in MIA PaCa-2 cells. GAPDH was used a loading control. SLM increases GPx4 protein expression. (**D**) SLM increases GPx4 activity in MIA PaCa-2 cells (means ± SEM, * *p* ≤ 0.05, unpaired *t*-test, *n* = 3).

In MIA PaCa-2 PDAC cells, P-AscH^−^ decreases clonogenic survival that is reversed when cells are treated with selenomethionine (Figure 4A). Similar results are seen when radiation treatment is combined with P-AscH^−^ (Figure 4A), demonstrating additional decreases in clonogenic survival that is reversed with the addition of selenomethionine. The same trends were seen with BODIPY fluorescence (Figure 4B), where P-AscH^−^ increased BODIPY fluorescence that was further enhanced with radiation. As seen in clonogenic survival, selenomethionine significantly reversed the P-AscH^−^- and radiation-induced increases in BODIPY oxidation (Figure 4B). One possible mechanism involved in these effects is selenomethionine-induced increases in phospholipid hydroperoxide glutathione peroxidase (GPx4). GPx4 is a selenoprotein that specifically terminates lipid peroxidation by removing lipid hydroperoxides and converting them to non-reactive alcohols. Selenomethionine significantly increases both the protein expression (Figure 4C) and activity (Figure 4D) of GPx4 in MIA PaCa2 cells compared to the control.

**Figure 5:**
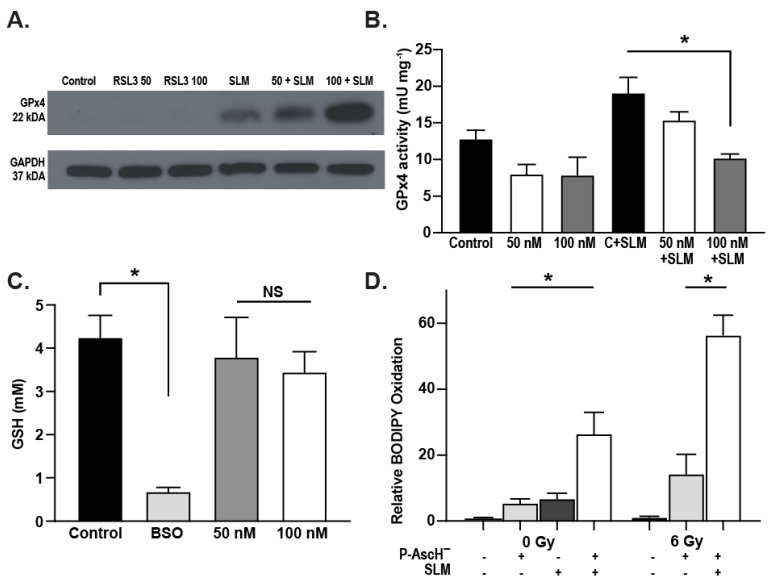
MIA PaCa-2 cells were treated with RSL3 (50–100 nM) with and without SLM (200 nM). (**A**) Representative Western blot image of GPx4 protein expression. GAPDH was used as a loading control. No changes were seen in GPx4 protein expression following RSL3 treatment. SLM increased GPx4 protein expression. (**B**) Increases in GPx4 activity induced by SLM-were reversed with RSL3 (means ± SEM, *n* = 3, * *p* ≤ 0.05 vs. RSL3 + SLM). (**C**) Concentrations of glutathione measured using L-buthionine-S,R-sulfoximine (BSO) as a positive control. BSO significantly decreased cellular GSH levels while no changes were seen after treatment with RSL3 (means ±SEM, * *p* ≤ 0.05, NS = not significantly different, *n* = 3). (**D**) Cells treated with or without 1 mM P-AscH^−^ (1 h, 10 picomoles cell^−1^) and RSL3 (50 nM) were radiated at ± 6 Gy, incubated with BODIPY C-11 for 30 min and evaluated under confocal microscopy. The combination of P-AscH^−^ and RSL3 significantly increased BODIPY oxidation in the absence and presence of radiation (means ± SEM, * *p* ≤ 0.05, *n* = 3).

To further investigate the mechanism, we used RAS-selective lethal 3 (RSL3), a compound that is known to inhibit GPx4 activity though direct binding, thereby disrupting redox homeostasis [33,34]. Inhibiting GPx4 expression, RSL3 is also known as a ferroptosis inducer, which has been demonstrated in several cancer types [34,35,36]. In MIA PaCa-2 cells, RSL3 (50 and 100 nM) did not affect GPx4 protein expression, but the addition of selenomethionine alone and in combination with RSL3 did increase expression (Figure 5A). Most importantly, RSL3 treatment reversed the selenomethionine-induced increases in GPx4 activity (Figure 5B). As previously observed by Yang et al., RSL3 did not decrease cellular GSH levels (Figure 5C) [34]. However, BODIPY fluorescence was increased with the combination of P-AscH^−^ and RSL3, suggesting that inhibiting GPx4 leads to increased lipid peroxidation (Figure 5D).

## 4. Discussion

Radiation plays a significant role as an adjuvant in treating locally advanced PDAC. Despite advances in radiation, e.g., stereotactic body radiation therapy and intensity-modulated radiation therapy, the ability to increase the radiation dose to more effective ranges while minimizing toxicity is still limited [7]. Our laboratory has previously published studies showing that P-AscH^−^ increases the radiosensitivity of cancer cells, leading to an improved tumoricidal effect of radiation [12,17,18,20]. Additionally, our research has suggested that there may be a protective factor of P-AscH^−^ on normal tissue [17,37]. This is the first study demonstrating the radioprotection of P-AscH^−^ treatment on intestinal tissue in humans. Consistent with the previous study where P-AscH^−^ mitigated the damage of radiation, our mouse model also showed a partial reversal of collagen deposition and villous blunting [17]. Furthermore, we delineate that this protection may be due to differences in oxidative stress where P-AscH^−^ increases lipid peroxidation in tumor cells but not in non-tumorigenic cell lines.

Previous studies have shown that the increased sensitivity of PDAC cells to P-AscH^−^ may be due to a H_2_O_2_ mechanism, suggesting the difference lies in cancer cells having less catalase activity and a lower capacity to remove H_2_O_2_ [18,32,38,39]. We further postulate that the differences in lipid peroxidation may explain the protection that P-AscH^−^ has on normal tissue. Lipid peroxidation typically occurs in cell membranes as reactive oxygen species (ROS) remove electrons, thereby forming lipid-based free radicals which then trigger downstream effects including ferroptosis. Radiation not only induces direct DNA damage but increases ROS, leading to free radical-mediated lipid peroxidation [40]. Our experiments have demonstrated that cancer cells have increased lipid peroxidation upon treatment with P-AscH^−^ in the setting of radiation; however, non-tumorigenic cells show minimal changes in oxidative stress. This is consistent in our in vivo mouse studies where tumor samples had increased 4-HNE protein expression with the combination of radiation and P-AscH^−^, while there was no significant change in the jejunal samples.

Selenoproteins, like glutathione peroxidases, play an active role in the removal of both hydroperoxides through GPx1 and phospholipid hydroperoxides via GPx4 [30,41,42]. The blunting of free radical-mediated lipid peroxidation by GPx4 is assisted upstream by donor antioxidants tocopherol and ascorbate [30,43]. Here, we demonstrate that the addition of selenium increases the activity of GPx4 in cancer cells, which leads to the reversal of lipid peroxidation seen with P-AscH^−^ treatment. There is also an increase in surviving fraction when cells are treated with the combination of selenomethionine and P-AscH^−^. The GPx4 inhibitor RSL3 has been shown to induce cell death and ROS accumulation [44,45]. Our current study demonstrates comparable results in MIA PaCa-2 cells where RSL3 reverses the effect of selenomethionine on GPx4 activity and increases lipid peroxidation alone and in combination with P-AscH^−^ and radiation.

## 5. Conclusions

In conclusion, our current study adds data demonstrating that P-AscH^−^ acts as a radiosensitizer of tumors, specifically PDACs. Most importantly, we have now shown in cell cultures, animal models, and humans that P-AscH^−^ induces radioprotection in normal cells/tissues. This radiosensitivity and radioprotection may be in part due to differences in oxidative stress between cancer and normal cells.

## Data Availability

Data is contained within the article and Supplementary Material.

## Supplementary Materials

The following supporting information can be downloaded at: https://www.mdpi.com/article/10.3390/antiox13030361/s1, Figure S1: Catalase protein expression and activity.
