# Supplementary material for: Pharmacologic Ascorbate Radiosensitizes Pancreatic Cancer but Radioprotects Normal Tissue: The Role of Oxidative Stress-Induced Lipid Peroxidation
Source: Antioxidants (Basel). 2024 Mar 18;13(3):361. doi: 10.3390/antiox13030361 (PMC10967795; doi:10.3390/antiox13030361)
Supplement: Supplementary file 1 [file antioxidants-13-00361-s001.zip › antioxidants-2864736-supplementary.pdf]

Supplemental Materials For:

Pharmacologic Ascorbate Radiosensitizes Pancreatic Cancer but Radioprotects Normal Tissue: The Role of Oxidative Stress-induced Lipid Peroxidation

Gloria Y. Chen[1*], Brianne R. O'Leary[1,2*], Juan Du[1,2], Rory S. Carroll[1], Garett J. Steers[1], Garry R. Buettner[2], and Joseph J. Cullen[1,2]

From the Departments of Surgery[1] and Free Radical and Radiation Biology Division, Department of Radiation Oncology[2], The University of Iowa Carver College of Medicine, Iowa City, IA, USA.

**Contributed equally to the authorship of this work.

Address correspondence to: Joseph J. Cullen, M.D.
1528 JCP, University of Iowa Hospitals and Clinics, Iowa City, IA 52242
joseph-cullen@uiowa.edu
W: (319) 353-8297, Fax: (319) 356-8378

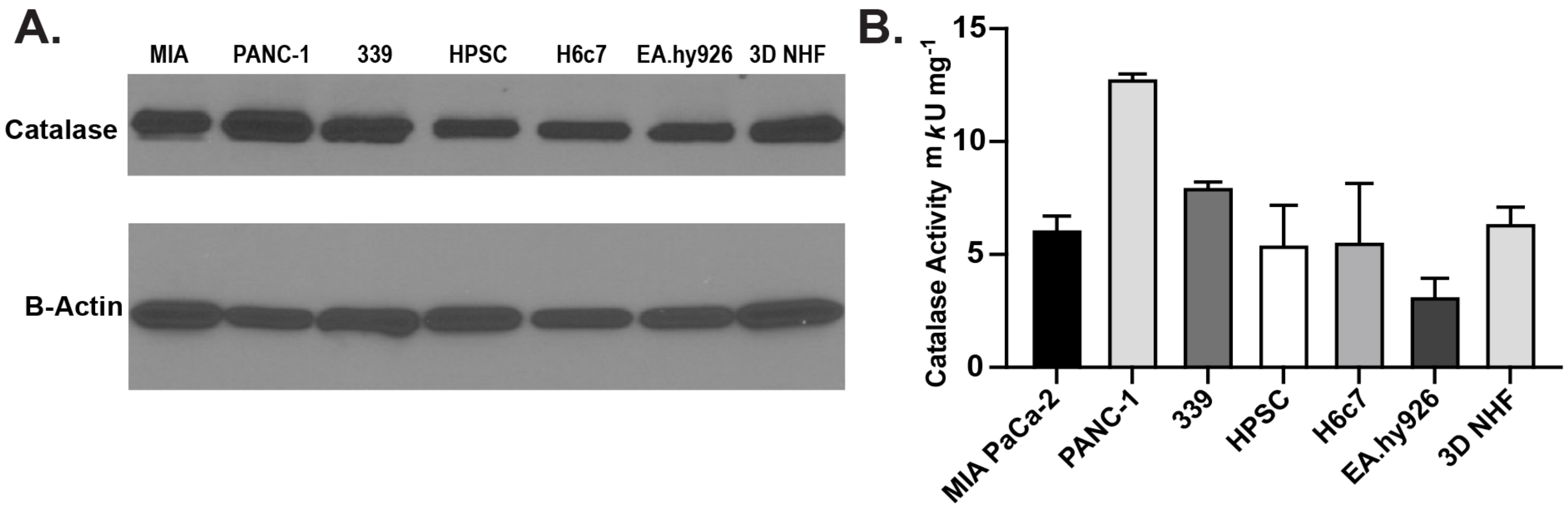

Supplemental Figure S1. Catalase protein expression and activity.

A. Representative Western blot image of catalase protein expression in various PDAC and normal pancreas cell lines (MIA PaCa-2, PANC-01, PDX-339, HPSC (human pancreatic stellate cells), H6c7 (non-tumorigenic pancreatic epithelial cells), EA.hy926 (human umbilical vein cells), and 3D NHF (3-day normal human fibroblast cells).

B. Mean catalase activity levels in the same cell lines, means ± SEM, n = 3.
